# Tracking immune dysregulation in COVID-19: lymphocyte dynamics from hospitalization to recovery

**DOI:** 10.1590/1414-431X2025e14960

**Published:** 2025-11-14

**Authors:** G.S. Eburneo, M.B. Sousa, M.K.C. Brunialti, S.S. Santos, J.G.D. Silva, P.R.A. Ferreira, N.C.J. Bellei, J.S.O. Arakaki, G.G.F. Leite, R. Salomao

**Affiliations:** 1 Disciplina de Infectologia, Departamento de Medicina, Escola Paulista de Medicina, Universidade Federal de São Paulo, São Paulo, SP, Brasil; 2 Disciplina de Pneumologia, Departamento de Medicina, Escola Paulista de Medicina, Universidade Federal de São Paulo, São Paulo, SP, Brasil

**Keywords:** COVID-19, Immune dysregulation, Immunophenotyping, Lymphocyte exhaustion, Lymphocyte activation

## Abstract

A hallmark of COVID-19 patients is the reduction of the lymphocyte population accompanied by activation, senescence, and exhaustion markers. We investigated patients admitted to hospital wards who either recovered after a short hospitalization or progressed to critical illness. Patients (n=48) were recruited between May and September 2020; 19 healthy volunteers were enrolled as controls. Blood samples were collected on days 0, 3, and 7 of hospitalization and around 30 days after discharge (convalescence sample, CS30). Lymphocyte counts and extended immunophenotyping were performed by flow cytometry and analyzed using conventional and stochastic methods. At D0 and D7, total lymphocytes, natural killer cells, T cells, TCD4 cells, and TCD8 cells were lower in patients than in volunteers but were restored at CS30. The stochastic analysis identified 11 distinct clusters of lymphocytes, nine of them with significant differences between patients and healthy controls. Clusters of TCD8+ memory cells showing activation, senescence, and exhaustion were increased in patients during hospitalization and in the convalescence samples. In contrast, clusters 5 (TCD4+ Central Memory exhausted activated) and 7 (TCD4+ Central Memory exhausted) were decreased in patients during the disease compared to healthy controls. Overall, the conventional flow cytometry analyses corroborated the findings from the stochastic analysis, showing that effector memory (EM) and TEMRA subsets exhibited sustained markers of exhaustion and senescence, particularly in TCD8+ cells. Our findings reinforce lymphopenia, T cell activation, senescence, and exhaustion as essential immunological features of COVID-19; while cell counts fully recovered, lymphocytes remained dysfunctional in convalescent samples.

## Introduction

Since its first report, the coronavirus disease 2019 (COVID-19) pandemic has caused an unprecedented global public health and social burden, exposing the critical consequences of socioeconomic inequalities on health outcomes (1,2). By August 2023, the numbers of COVID-19 cases exceeded 700 million, which caused almost 7 million deaths (3). Patients who survived COVID-19 may experience persistent symptoms and disabilities after hospital discharge, a condition known as post-acute or long-COVID (4).

The clinical presentation of COVID-19 is highly heterogeneous, ranging from asymptomatic (or presymptomatic) cases to mild, moderate, severe, and critical disease (3). Several studies have investigated clinical symptoms, routine laboratory changes, and biological markers that predict patient outcomes. Poor outcomes have been associated with decreased lymphocyte and platelet counts, along with elevated levels of C-reactive protein, D-dimer, and creatinine, among other factors (5). Lymphopenia and reductions in lymphocyte populations (T, B, and NK) have been associated with the clinical severity of COVID-19 (6), with a marked dichotomy in the recovery of lymphocyte counts between survivals and non-survivals (7). In our analysis, patients stratified by clinical course showed that those with a deteriorating trajectory exhibited persistent lymphopenia, neutrophilia, and a consequent higher neutrophil-to-lymphocyte ratio (NLR) (8).

In addition to reduced circulating cell numbers, T lymphocytes in COVID-19 patients have been reported to exhibit markers of activation, senescence, and exhaustion (9-11), with more accentuated dysregulation observed in severe cases (12).

Despite substantial evidence of lymphocyte dysregulation in COVID-19, data on prospective and longitudinal evaluations, particularly involving post-discharge samples, remain limited. During the pandemic, patients with COVID-19 were assisted in the inpatient or outpatient settings depending on clinical presentation and potential risk factors for severe disease (13). Patients with critical illness were referred to the ICU, while those with moderate disease were admitted to hospital wards.

The purpose of this study was to track lymphocytes' immune dysregulation in COVID-19 patients by evaluating markers of cellular activation, exhaustion, and senescence by flow cytometry. We focused on moderately ill patients admitted to the hospital wards and presenting clinical recovery and short hospitalization or clinical deterioration and need for intensive care unit (ICU) support (8). Samples were obtained prospectively during the clinical course of the disease and after hospital discharge, allowing us to compare lymphocyte changes during disease and convalescence in patients with distinct outcomes.

## Material and Methods

### Study design, population, and setting

This study was conducted in accordance with ethical principles and was approved by the Research Ethics Committee, under approval number 4.018.308. All participating volunteers signed the Informed Consent Form.

The cohort consisted of patients over 18 years of age diagnosed with COVID-19, with a positive polymerase chain reaction (PCR) testing for SARS-CoV-2 using nasopharyngeal swabs, who were admitted to Hospital São Paulo, the University Hospital of the Federal University of São Paulo, Brazil, between May and September 2020. Patients presented with moderate to severe disease, defined according to the World Health Organization (WHO) and the National Institutes of Health (NIH) guidelines (14,15). Healthy volunteers, matched for gender and age with the patients and with negative results in PCR and serology tests for COVID-19, were included as a control group. Clinical and epidemiological data have been published elsewhere (8). For this study, kidney transplant recipients were excluded because of the effects of the underlying disease and immunosuppressive therapy on the evaluated lymphocyte dysfunctions.

Patients were classified as critical if they presented clinical deterioration and organ dysfunctions requiring intensive treatment and as non-critical if they presented clinical recovery and hospital discharge.

Samples were collected at different time points: at admission (Day 0), on the third day of hospitalization (D3), on the seventh day of hospitalization (D7), and in the convalescence samples (CS30), which were collected one month after hospital discharge. Blood samples (30 mL) were collected in EDTA tubes. A 500-µL aliquot was separated for lymphocyte counting, while the remainder of the samples was used for the separation of peripheral blood mononuclear cells (PBMC) using the Ficoll gradient method (Cytiva Life Sciences, Sweden).

### Flow cytometry

The samples were processed and analyzed using the FACSCalibur flow cytometer for lymphocyte cell counts (TruCount method), and the LSR Fortessa for extended immunophenotyping (both from BD Biosciences, USA).

### TruCount

The absolute number and percentage of NK cells, B lymphocytes, T cells, and T cell subpopulations (TCD4+ and TCD8+) were determined in whole blood using the TruCount method (BD Biosciences). Cells were stained using two separate tubes. The first tube contained MultiTest monoclonal antibodies for CD45- PerCP, CD3-FITC, CD4-APC, and CD8-PE and the second tube included MultiTest monoclonal antibodies for CD19-APC, CD3-FITC, CD16/CD56-PE, and CD45-PerCP. The samples were then incubated for 15 min in the dark at room temperature. Red blood cells were lysed using 450 µL of FACS Lysing Solution diluted 1:10. All reagents were from BD Biosciences. Samples were acquired and analyzed using MultiSet software in a FACSCalibur flow cytometer (BD Biosciences).

### Extended immunophenotyping

Immunophenotyping of T lymphocytes was conducted in peripheral blood mononuclear cells (PBMC) to evaluate the expression of markers of differentiation, activation, senescence, and exhaustion. PBMC samples were resuspended to 3×10^5^ cells per cytometry tube and washed. A staining pool of antibodies was prepared for the samples using Brilliant Stain Buffer PLUS (BD Biosciences) and added to the samples, as specified in Table 1. After the addition of 2 mL of MACS buffer, samples were centrifuged at 800 *g* for 5 min at 4°C. The cells were fixed with 1% paraformaldehyde and incubated in the dark on ice for 30 min, followed by washing with MACS buffer and resuspended in 300 µL of MACS buffer. Thirty thousand events were acquired in a SSC × FSC gate compatible with lymphocytes. To perform compensation, BD™ CompBeads were used. In each compensation tube, positive beads, negative beads, and a specific antibody were added, except for the “blank” tube, which contained no staining.

**Table 1:** Markers and specifications for advanced immunophenotyping.

Target	Fluorophore	Clone	TITER (µL)	Purpose
HLA- DR	PE- CF594	G46-6	1	Activation
CD3	APC- Cy7	SK7	1	T cell lineage
CD57	BV605	QA17404	1	Senescence
PD-1	BV711	EH12.1	1	Exhaustion
CD4	FITC	RPA- T4	2	CD4 T cell lineage
CCR7	BV421	2-L1-A	2	Memory subpopulations or naive
CD8	PerCP	SK1	8	CD8 T cell lineage
CD38	PE	HIT2	8	Activation
CD45RA	APC	HI100	8	Memory subpopulations or naive

The “fluorescence minus one” (FMO) method, staining with all fluorophores except one per tube, was used to set the gates for HLA-DR, CD38, CCR7, CD45RA, CD57, and PD-1 (Supplementary Figure S1).

### Conventional analysis

For conventional analysis, flow failures, debris, and cellular artifacts were excluded. The strategy of Brummelman et al. (16) was employed to remove dead cells or doublets that could generate erroneous clusters. To characterize the stages of memory differentiation in T lymphocytes, a graph was generated based on morphology, size, and complexity (SSC × FSC) to highlight the T lymphocyte population based on CD3 positivity. The TCD4+ and TCD8+ subpopulations were selected, and naive/memory differentiation was analyzed using the markers CD45RA and CCR7. Markers of activation (HLA-DR and CD38), exhaustion (PD-1), and senescence (CD57) were analyzed in all subpopulations of TCD4 and TCD8 (Supplementary Figure S2).

### Stochastic analysis

For stochastic analysis, 3,000 TCD3+ cells were randomly selected using the DownSample plugin in FlowJo™ software, version 10.17 (BD). The data files were concatenated and clustered using the FlowSOM plugin (17). The AutoGateCategorical plugin identified individual subjects within the clusters. Fluorescence data from FlowSOM was used to generate a heatmap with z-score calculations. Cluster visualization was performed using a t-SNE plot in FlowJo.

### Statistical analysis

Data analyses were performed in the R environment (version 4.4.0) for Windows. Normality of continuous variables was assessed using the Shapiro-Wilk test and visualized with Quantile-Quantile (Q-Q) plots and histograms. Q-Q plots and histograms were employed as complementary tools to inspect deviations from normality, providing both numerical and graphical assessments. Homogeneity of variances was evaluated using Levene's test. Continuous variables with a normal distribution and homogeneous variances were analyzed using one-way ANOVA followed by Tukey's *post hoc* test for multiple group comparisons. For continuous variables with a normal distribution but unequal variances, Welch's ANOVA with Games-Howell *post hoc* tests were applied. Non-normally distributed data were analyzed using the Kruskal-Wallis test with Dunn's *post hoc* tests for multiple groups. Categorical variables were analyzed using the chi-squared test, with Fisher's exact test applied when expected frequencies were below 5. Descriptive statistics are reported as means±SD for normally distributed data and as medians with interquartile range (IQR) for non-normally distributed data. Statistical significance was set at a P-value ≤0.05. Graphs were generated using the ggplot2 and pheatmap packages.

## Results

### Baseline characteristics and clinical outcomes

The study included 48 patients with COVID-19 and 19 healthy volunteers, matched for age and gender. The mean age of the patients was 59 years, ranging from 21 to 76 years old, and most of the patients were male (n=30, 63%). The median hospital stay was 9 days (interquartile range [IQR]: 5-16) and the median duration of symptoms before admission was 7 days (IQR: 5-10). The most frequently reported symptoms were fever (n=32; 67%), cough (n=36; 75%), shortness of breath (n=31; 65%), and diarrhea (n=14; 29%). Among the 48 patients, 14 (29%) progressed to clinical deterioration and critical illness, while 34 (71%) presented clinical recovery and were classified as non-critical. Detailed epidemiological, clinical, and routine laboratory data for patients with critical illness and those who recovered are presented in Table 2.

**Table 2:** Epidemiologic, clinical, and routine laboratory information.

	Global (n=48)	Critical (n=14)	Non-critical (n=34)	P-value*
Demography				
Sex (male)	30 (63%)	9 (64%)	21 (61%)	≥0.9
Age	59 (13)	64.93 (7.8)	55.98 (14.2)	**0.034**
Hospital days	9 (5-16)	23 (16-35.5)	6 (4-9.75)	**≤0.0001**
Mortality	4 (8.3%)	4 (28.6%)	0 (0%)	**0.005**
Admission data				
Fever	32 (67%)	8 (57%)	24 (70%)	0.4
Cough	36 (75%)	10 (71%)	26 (76%)	0.7
Shortness of breath	31 (65%)	11 (78%)	20 (58%)	0.2
Diarrhea	14 (29%)	5 (35%)	9 (26%)	0.5
Cardiac rate	88 (16)	86.93 (15.6)	88.21 (16.1)	0.8
Respiratory rate	24 (20-26)	22.5 (20-24)	24 (20-26.5)	0.5
SpO_2_	94 (91-95)	94 (91.5-94)	94 (91.2-95)	0.6
Body mass index	28 (24-33)	24.85 (24.2-27.8)	29.25 (24.7-33)	**0.03**
SOFA score	1.6 (1.6)	2 (0.25-3)	1 (0-2)	0.2
Comorbidities				
Cardiac disease	12 (25%)	3 (21%)	9 (26%)	0.7
COPD	9 (19%)	3 (21%)	6 (17%)	0.8
Diabetes	20 (41.6%)	7 (50%)	13 (38%)	0.5
CKD	4 (8.3%)	3 (21%)	1 (2%)	**0.04**
Hypertension	27 (56.3%)	10 (71%)	17 (50%)	0.2
Obesity	7 (15%)	1 (7.1%)	6 (17%)	0.3
Charlson index	3 (1-5)	4.5 (3-5.75)	2 (1-4)	**0.009**
Sample collection timelines				
Days from symptoms onset to hospital admission	7 (5-10)	6.5 (3.5-7)	7 (6-10.7)	**0.05**
Hospital discharge to convalescent sample	28 (15-53)	21.5 (11.7-39.2)	28 (19.5-57.5)	0.1
Laboratory admission				
Lymphocytes, cells/µL	1,027 (439)	845.8 (338.1)	1101.3 (458.5)	**0.04**
Neutrophils, cells/µL	4,700 (3,578-7,488)	7,058 (4,441.5-10,616.5)	4,500 (3,304.5-6,322.5)	**0.01**
Monocytes, cells/µL	294 (226-503)	430 (284-581.2)	281 (223.2-464.2)	0.1
NLR	5.2 (4)	10.4 (5.7)	5.8 (4.7)	**0.002**
Platelets, cells/µL	190,500 (153,500, 236,000)	188,500 (141,250-240,250)	190,500 (157,750-230,000)	0.7
Hemoglobin, g/dL	13.1 (1.8)	12.31 (2.2)	13.46 (1.6)	0.1
Creatinine, mg/dL	39 (25-66)	0.89 (0.73-1.2)	0.87 (0.72-1.0)	0.5
CRP, mg/L	85 (57-165)	111.8 (67.32-218)	80.69 (51.7-122.6)	0.2
D-dimer, µg/mL FEU	1.25 (0.74-2.6)	2.18 (0.79-4.2)	1.15 (0.70-2.2)	0.2

Normally distributed variables are reported as means±SD and compared by Student's *t*-test. Non-normally distributed variables are reported as median and 25th and 75th percentiles and compared by Mann-Whitney. SpO_2_: oxygen saturation; SOFA score: Sequential Organ Failure Assessment; CKD: chronic kidney disease: NLR: neutrophil-to-lymphocyte ratio; COPD: chronic obstructive pulmonary disease; CRP: C-reactive protein. Values in bold are significantly different. *Critical *vs* Non-critical.

### Lymphocyte counts in COVID-19 patients during disease progression

**Figure 1:**
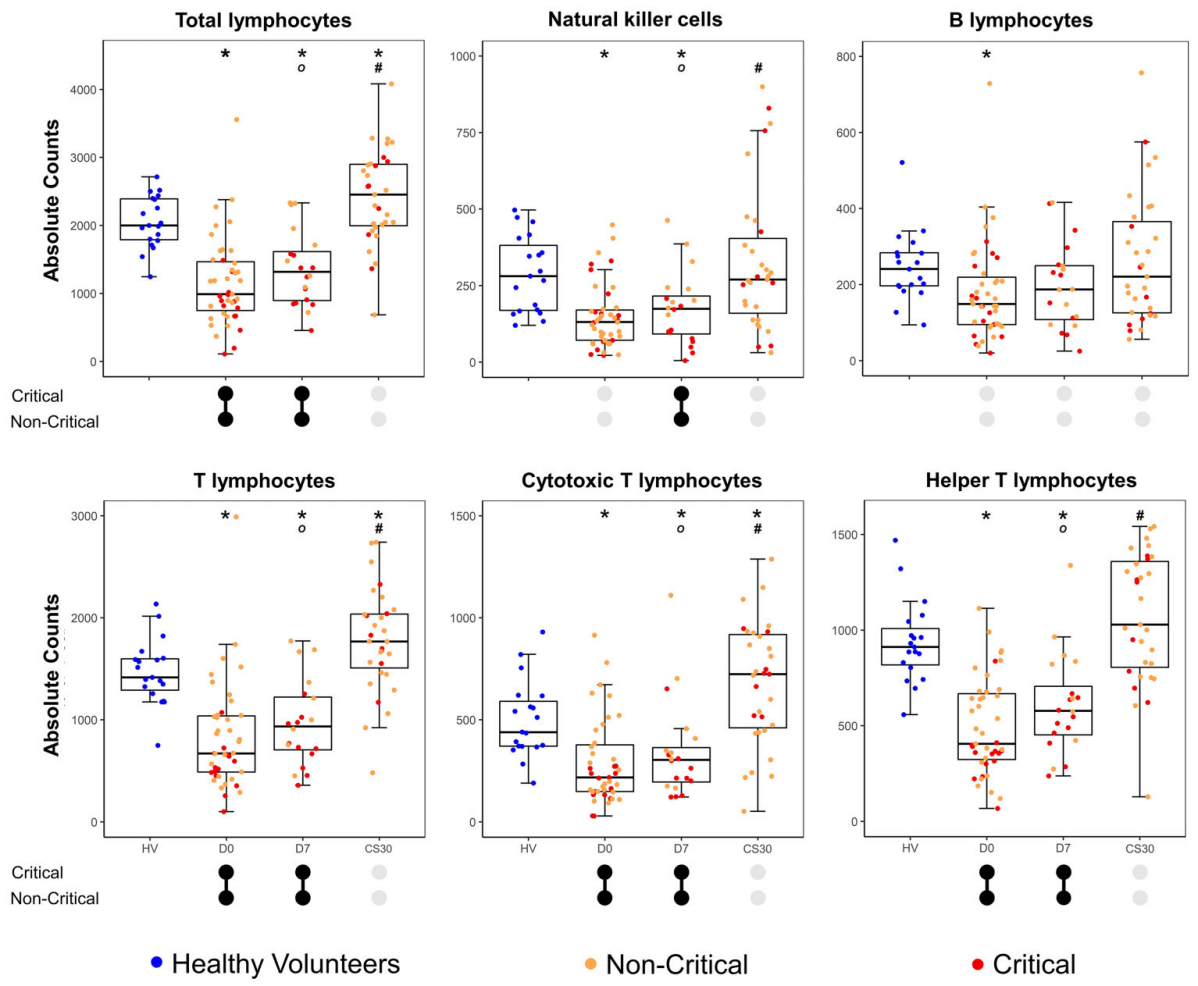
Box-and-whisker plots summarizing absolute lymphocyte counts across different populations and time points day (D) 0, D7, and convalescence sample (CS30). Each dot represents an individual sample. The box represents the interquartile range (IQR), with the central line indicating the median, and whiskers extending to the minimum and maximum values within 1.5 × IQR. *P≤0.05, compared to HV (Mann-Whitney U test). Comparisons among patients' follow-up samples (D0, D7, and CS30) were conducted using the Kruskal-Wallis one-way ANOVA with Dunn's *post hoc* test (^#^P<0.05, vs D0; °P<0.05, *vs* CS30). Connected black dots indicate significant differences between critical and non-critical patients at the same time point, while gray disconnected dots represent non-significant differences.

The absolute counts of lymphocyte populations were evaluated in healthy volunteers and COVID-19 patients at three time points: admission (D0), day 7 of hospitalization (D7), and around one month after discharge (CS30). These measurements were used to investigate changes during disease progression and recovery (Figure 1 and Supplementary Table S1).

Significant differences in absolute cell counts were observed between healthy volunteers and COVID-19 patients, as well as between patients with distinct clinical outcomes. At D0 and D7, total lymphocyte counts, natural killer (NK) cells, T cells, TCD4 cells, and TCD8 cells were significantly lower in patients compared to healthy volunteers, with a restoration in the cell counts at convalescence (CS30) (Figure 1). B-lymphocytes were less affected, with reduced counts only at admission (D0). No significant differences were found in the percentages of any cell population compared to the control group.

Patients who progressed to critical illness exhibited lower counts of total lymphocytes (D0 and D7), T cells (D0 and D7), TCD4 cells (D0 and D7), TCD8 cells (D0 and D7), and NK cells (D7) than patients with non-critical illness, with no differences in convalescent samples (CS30) (Figure 1 and Supplementary Table S1). Longitudinal analysis revealed an increase in total lymphocytes, T cells, TCD4 cells, TCD8 cells, and NK cells in convalescent samples for both groups (Supplementary Table S1).

### Markers of T lymphocytes activation, senescence, and exhaustion

#### Unsupervised analysis using FlowSOM

**Figure 2:**
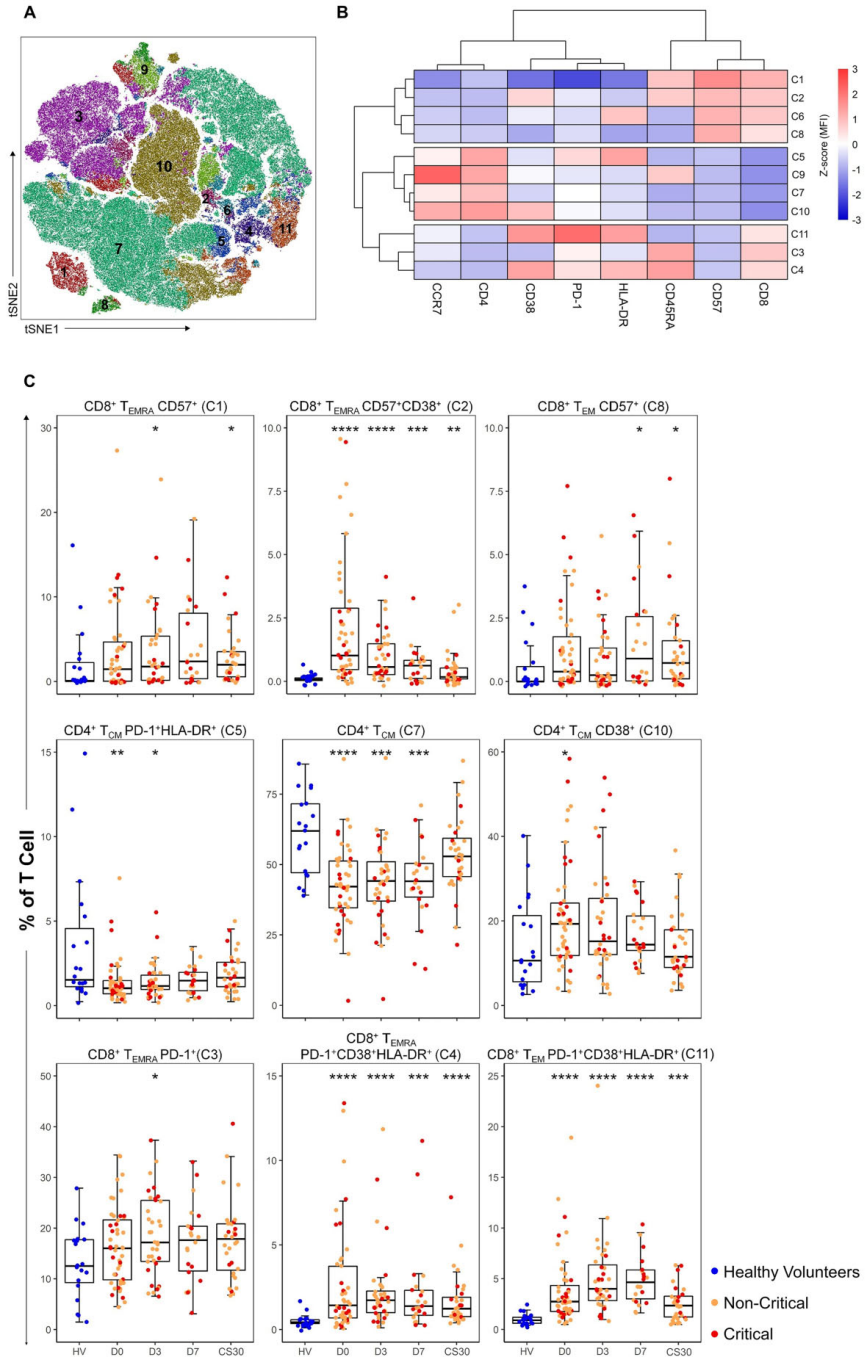
t-SNE projection, marker intensity, and cluster distribution of T cell populations. **A**, t-SNE projection of T cell clusters identified by FlowSOM clustering across all patients and time points. **B**, Heatmap showing the mean fluorescent intensity (MFI) of various markers in each T cell cluster, with column-scaled z-scores. Hierarchical clustering is represented by dendrograms on the left (clusters) and top (markers), illustrating the similarity between clusters and markers. **C**, Box-and-whisker plots depicting the median (interquartile range (IQR)) percentage of T cells from each cohort in each FlowSOM cluster. *P<0.05, **P<0.01, ***P<0.001, ****P<0.0001 compared to HC (two-sided Mann-Whitney U test). All statistical results are detailed in Supplementary Table S3.

The stochastic analysis identified 11 distinct clusters of lymphocytes, including seven clusters of TCD8+ lymphocytes and four of TCD4+ lymphocytes, based on the expression of differentiation, activation, senescence, and exhaustion markers, as illustrated in Figure 2A and B, and characterized in Supplementary Table S2. Among these, clusters 7, 3, and 10 were the most representative in terms of cell percentages.

Significant differences between patients and healthy controls were observed in nine clusters (Figure 2C). Specifically, clusters 2 (CD8+ CD45RA+ CD57+ CD38+), 4 (CD8+ CD45RA+ PD-1+ CD38+ HLA-DR+), and 11 (CD8+ PD-1+ CD38+ HLA-DR+) were increased in patients during hospitalization and in the convalescence samples. In contrast, clusters 5 (CD4+ CCR7+ PD-1+ HLA-DR+) and 7 (CD4+ CCR7+) were decreased in patients compared to healthy controls during the disease, but did not show differences in convalescent samples.

No significant differences in cluster percentages were observed between patients who progressed to critical illness and those who recovered, except for cluster 10 on D3 and clusters 6 and 11 in convalescent samples (Supplementary Table S2).

#### Conventional flow cytometry analyses

Overall, the conventional flow cytometry analyses corroborated the findings from the stochastic analysis, with most changes observed in memory T cell populations.

**Figure 3:**
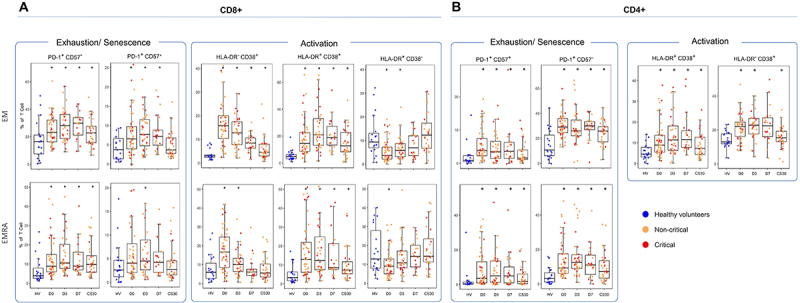
Immunophenotyping of memory cells in COVID-19 patients at admission and during follow-up. Memory cells are characterized as Central Memory (CD45RA- CCR7+), Effector Memory (EM) (CD45RA- CCR7-), and Effector Memory RA (EMRA; CD45RA+ CCR7). Cellular activation is marked with HLA-DR and/or CD38, and exhaustion and senescence are marked with PD-1 and CD57, respectively. Boxplot showing median (interquartile range (IQR)) of memory subpopulations in CD8 cells (**A**) and in CD4 cells (**B**) from patients and healthy volunteers. *P<0.05 compared to healthy volunteers (Mann-Whitney U test).

Among T CD8 cells, effector memory cells showed an increased HLA-DR and CD38 (CD45RA- CCR7- CD38+ HLA-DR+) expression in patients at all time points. While CD38 (CD38+ HLA-DR-) expression alone was increased, HLA-DR expression alone (CD38- HLA-DR+) was decreased in patients compared to controls. An increase in the expression of PD-1 (CD45RA- CCR7- PD-1+ CD57) and co-expression of PD-1 and CD-57 (CD45RA- CCR7- PD-1+ CD57+) was also observed on D0, D3, and D7, but not on CS30 samples. TCD8+ RA Effector Memory (TEMRA) cell showed increased expression of CD38 and HLA-DR, and again, a lower expression of HLA-DR alone. Similarly, we observed a significant increase in PD-1 (CD45RA+ CCR7- PD-1+ CD57-) at all time points (Figure 3A). Central memory cells exhibited increased CD38 (CD38+ HLA-DR-) and lower HLA-DR alone (CD38- HLA-DR+) during the acute phase compared to controls. Over time, there was a decrease in the expression of HLA+CD38+ on TCD8+ memory cells in patients, an effect resulting from the kinetics in non-critical patients (Supplementary Table S3).

Among T CD4 cells, effector memory cells showed increased HLA-DR and CD38 (CD45RA- CCR7- CD38+ HLA-DR+) expression in patients at all time points. Additionally, elevated expression of PD-1 (CD45RA- CCR7- PD-1+ CD57-) and co-expression of the PD-1 and CD-57 (CD45RA- CCR7- PD-1+ CD57+) were also observed at all time points. Similarly, in the TEMRA cell, a significant increase in PD-1 and CD57 co-expression (CD45RA+ CCR7- PD-1+ CD57+) and PD-1 expression alone was noted (Figure 3B). In contrast, TEMRA cells double-negative for HLA-DR and CD38 showed an increase in frequency. Changes were less pronounced in central memory cells (Supplementary Table S3).

Notably, the alterations in CD4+ T cell populations persisted throughout the disease course and into convalescence. Furthermore, these changes did not differ significantly between patients who progressed to critical illness and those who recovered (Supplementary Table S3).

## Discussion

Hematologic changes have been reported in early COVID-19 studies (18), with reduced lymphocyte counts and expressing markers of activation, senescence, and exhaustion. In this study, we examined lymphocyte counts and immunophenotyping in hospitalized patients who either progressed to clinical resolution or experienced clinical deterioration. Additionally, convalescent samples from patients with the two clinical outcomes were evaluated. Importantly, while lymphopenia was resolved in convalescent samples, markers of immune dysfunction persisted, suggesting a longer-lasting impact on adaptive immunity.

One interesting aspect of the host response to COVID-19 is an exaggerated inflammatory response concomitant with a state of immunodeficiency. Following SARS-CoV-2 infection of lung epithelial cells, patients progressing to severe illness present excessive cell infiltration, systemic cytokine storm, pulmonary dysfunction, and widespread inflammation and multi-organ damage (reviewed in Tay et al. (19) and Vardhana and Wolchok (20)). This exacerbated response is accompanied by a dysfunctional adaptive response, which compromises the control of virus replication and predisposes to secondary infections.

Our study focused on lymphocytes immunophenotyping, finding that lymphocyte cell counts were dynamically altered during disease in COVID-19 patients, with total lymphocyte, NK cell, T cell, TCD4 cell, and TCD8 cell counts significantly reduced during hospitalization and subsequently restored in convalescent samples. Notably, despite similar clinical presentations at admission, patients who progressed to critical illness exhibited marked reductions in TCD4+ and TCD8+ counts, as well as NK cells, compared to those with clinical recovery, particularly at D0 and D7. This finding reinforces previous single-time-point reports associating lymphopenia with severe disease (6,12). Our results align with Zhou et al. (7), who reported distinct temporal changes in inflammatory markers and lymphocyte counts between survivors and non-survivors, underscoring the dynamic nature of immune alterations in COVID-19.

In addition to changes in T cell subset counts, our study revealed profound phenotypic alterations in TCD4+ and TCD8+ cell subsets, characterized by increased activation (HLA-DR+, CD38+), senescence (CD57+), and exhaustion (PD-1+) markers, consistent with De Biasi et al. (9) and Tay et al. (21). Thus, immunosuppression in COVID-19 patients may be due to both T cell depletion and dysfunction. A robust T cell-mediated adaptive response is crucial for effective viral clearance and disease control (22). Early HIV control is achieved through robust HIV-specific T cell responses, particularly TCD8+ cells, which is critical for managing the infection and preventing disease progression (23). CD57+ expression in T lymphocytes has been recognized as a marker of *in vitro* replicative senescence and of functional immune deficiency in patients with autoimmune disease, infectious diseases, and cancers (reviewed in Focosi et al. (24)). HIV-specific TCD8 cells expressing CD57 have been shown to produce cytokines but presented replicative senescence and antigen-induced apoptotic death of CD8+ T cells (25). The COVID-19 patients in our cohort presented increased CD57 co-expressed with PD-1. Programmed cell death protein 1 (PD-1) down-regulates the immune response and suppresses T cell inflammatory activity; in chronic infections, it leads to T cell exhaustion and impairs the ability of killing infectious cells (reviewed in Aghbash et al. (26)). In a single time-point study, De Biasi et al. (9) reported that COVID-19 patients expressed higher percentages of senescent/exhausted TCD4 and TCD8 cells (PD1+CD57+) (9), showing that this phenotype persisted and increased during disease and, for TCD4 cells, even in convalescent samples.

In our cohort, the frequency of TCD8+HLA-DR^+^CD38^+^ cells was higher in COVID-19 patients than in healthy controls, while HLA-DR^+^CD38^+^ cells were notably decreased. These findings broadly align with the study by Du et al. (27), who also reported elevated TCD8+HLA-DR^+^CD38^+^ cells in patients with mild, moderate, and especially severe disease. Interestingly, they did not find any significant differences in HLA-DR^+^CD38^+^ cells compared to healthy donors. Notably, Du et al. (27) demonstrated that the overall HLA-DR^+^CD38^+^ population can be further subdivided into HLA-DR^+^CD38^dim^ and HLA-DR^+^CD38^high^ subsets, with persistent elevation of the CD38ˆhi fraction strongly correlating with immune dysregulation and severe disease.

Our unsupervised clustering analysis identified 11 distinct T cell populations, nine of which differed significantly between patients and controls. Notably, three clusters (including memory TCD8+ cells expressing PD-1 or CD57) remained altered even in convalescent samples, reinforcing findings from Breton et al. (28) that T cell exhaustion persists post-infection. Interestingly, the three clusters showed concomitant markers of senescence/exhaustion (CD57/PD1) and cellular activation (HLA-DR/CD38).

Conventional flow cytometry analyses supported these findings, showing that EM and TEMRA subsets exhibited sustained markers of exhaustion and senescence, particularly in TCD8+ cells. Interestingly, while T cell counts were lower in critically ill patients, exhaustion and activation markers did not significantly differ between severe and moderate cases, in agreement with Sekine et al. (10), suggesting that T cell dysfunction is a hallmark of COVID-19 regardless of disease severity.

In conclusion, our findings reinforce lymphopenia, T cell activation, senescence, and exhaustion as key immune hallmarks of COVID-19, in agreement with previous reports (6,7,9) and add evidence that while cell counts show full recovery, lymphocytes remained dysfunctional in convalescent samples, highlighting the need for long-term immune monitoring in recovered patients. Altogether, these findings open a new strategy therapy for COVID-19. Adjuvant immune therapy for COVID-19 has usually been focused on blocking hyperinflammation (e.g., corticosteroids, IL-6 inhibitors, JAK inhibitors), as recommended by WHO guidelines (29). Hotchkiss and colleagues have proposed that targeting immunosuppression would also benefit these patients (30). In addition, a reduction in the occurrence of secondary nosocomial infections has been shown in a randomized, double-blind, placebo controlled trial of IL-7 in critically ill COVID-19 patients (31). Given the heterogeneity of immune responses in COVID-19, a personalized therapeutic approach that balances immune suppression and inflammation may offer the most effective strategy for improving patient outcomes.

This study has some limitations. First, the study was conducted in a single hospital, which may limit the generalizability of the findings. Although the hospital serves a large and diverse population in São Paulo, Brazil, validation in different geographic and clinical settings is necessary for a broader applicability of our results. Second, while we analyzed lymphocyte activation, senescence, and exhaustion markers, functional assays to assess immune cell activity were not performed. Additionally, the influence of treatments received by patients on immune responses could not be fully accounted for.

## Supplementary Materials

Supplementary Material Click to view [zip].

## Data Availability

All data generated or analyzed during this study are included in this published article.
