# Supplementary material for: Tracking immune dysregulation in COVID-19: lymphocyte dynamics from hospitalization to recovery
Source: Braz J Med Biol Res. 2025 Nov 14;58:e14960. doi: 10.1590/1414-431X2025e14960 (PMC12645439; doi:10.1590/1414-431X2025e14960)

Figure S1. Quadrant strategy based on FMO (fluorescence minus one). **A**, The gating sequence demonstrates the selection of singlets, lymphocyte morphology, T lymphocytes, and the separation of TCD4 and TCD8 subsets. The fluorescence interference in the FMO controls was used to determine the positivity thresholds for each specific marker. **B**, FMO control for CD45RA labeled with APC. **C**, FMO control for CD57 labeled with BV605. **D**, FMO control for CD38 labeled with PE-CF594. **E**, FMO control for CCR7 labeled with BV421. **F**, FMO control for PD-1 labeled with BV711. **G**, FMO control for HLA-DR labeled with PE.

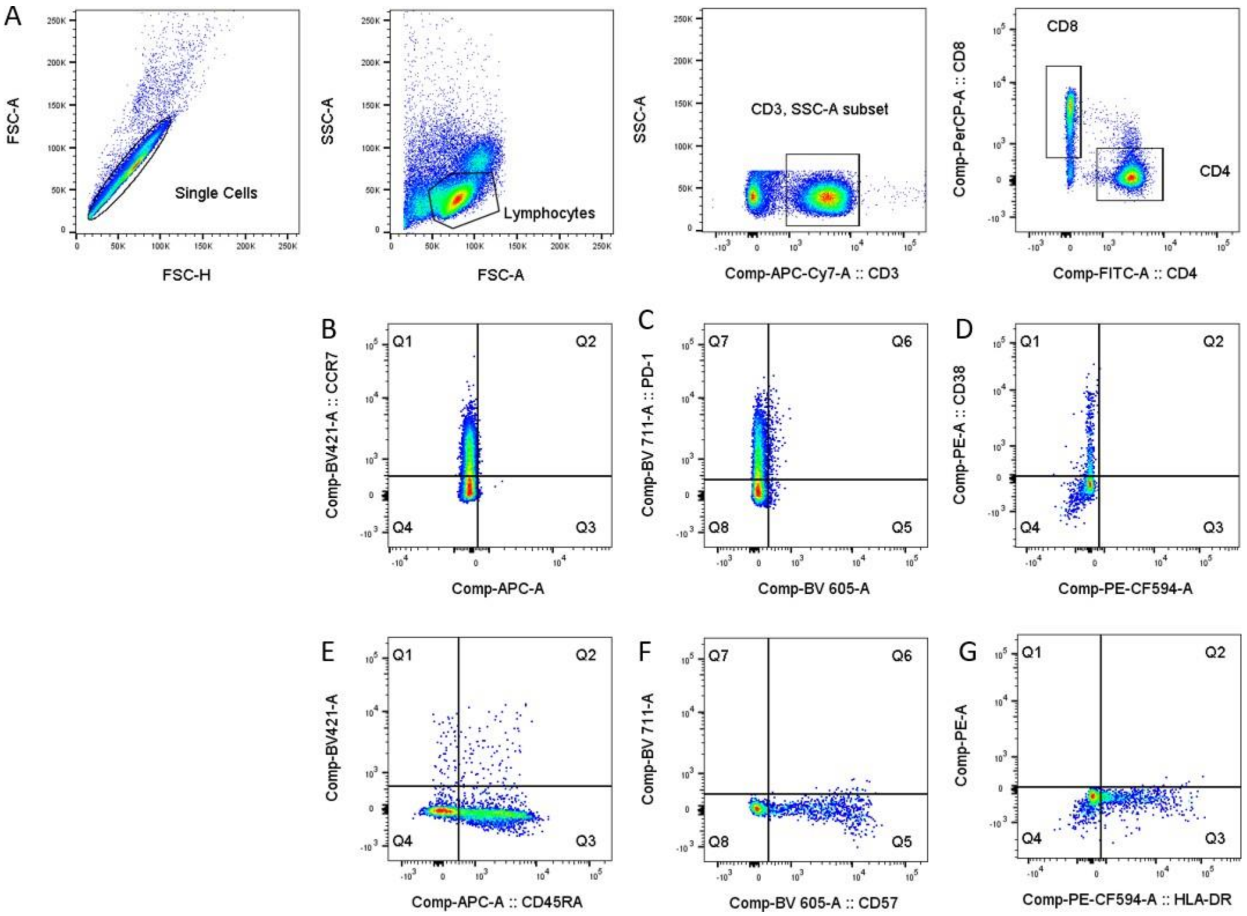

Figure S2. Quadrant strategy for activation, senescence, and exhaustion markers in memory subpopulations. **A**, Selection of TCD4 and TCD8 lymphocyte populations. **B**, Differentiation of subpopulations based on maturation state. **C**, Markers CD57/PD-1 (exhaustion and senescence) and CD38/HLA-DR (activation) applied to memory populations and naive T lymphocytes.

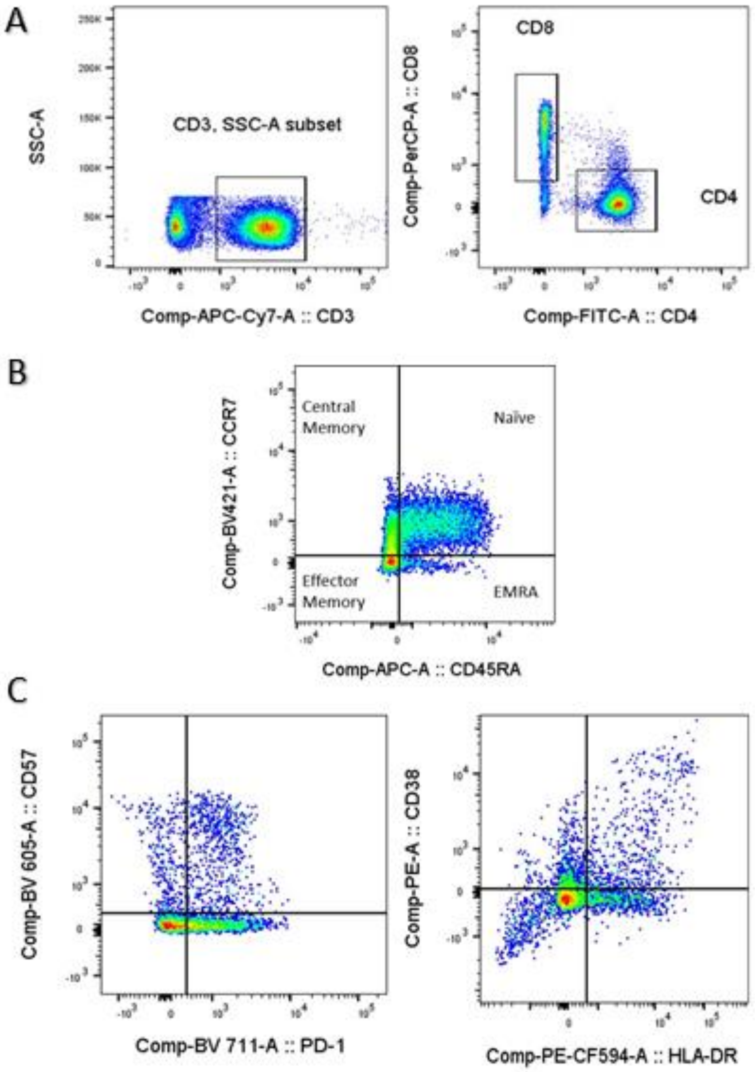

Supplement: Supplementary file 1 [file 1414-431X-bjmbr-58-e14960-suppl.zip › 14960 - Supplementary Figures.pdf]
